# Generalizable Framework for Atrial Volume Estimation for Cardiac CT Images Using Deep Learning With Quality Control Assessment

**DOI:** 10.3389/fcvm.2022.822269

**Published:** 2022-01-28

**Authors:** Musa Abdulkareem, Mark S. Brahier, Fengwei Zou, Alexandra Taylor, Athanasios Thomaides, Peter J. Bergquist, Monvadi B. Srichai, Aaron M. Lee, Jose D. Vargas, Steffen E. Petersen

**Affiliations:** ^1^Barts Heart Centre, Barts Health National Health Service Trust, London, United Kingdom; ^2^National Institute for Health Research Barts Biomedical Research Centre, William Harvey Research Institute, Queen Mary University of London, London, United Kingdom; ^3^Health Data Research UK, London, United Kingdom; ^4^Georgetown University School of Medicine, Washington, DC, United States; ^5^Montefiore Medical Centre, Bronx, NY, United States; ^6^Northeastern University, Boston, MA, United States; ^7^MedStar Heart and Vascular Institute, Washington, DC, United States; ^8^Veterans Affairs Medical Center, Washington, DC, United States; ^9^Georgetown University, Washington, DC, United States; ^10^The Alan Turing Institute, London, United Kingdom

**Keywords:** CT, left atrium, left atrial volume, quality control, deep learning

## Abstract

**Objectives:**

Cardiac computed tomography (CCT) is a common pre-operative imaging modality to evaluate pulmonary vein anatomy and left atrial appendage thrombus in patients undergoing catheter ablation (CA) for atrial fibrillation (AF). These images also allow for full volumetric left atrium (LA) measurement for recurrence risk stratification, as larger LA volume (LAV) is associated with higher recurrence rates. Our objective is to apply deep learning (DL) techniques to fully automate the computation of LAV and assess the quality of the computed LAV values.

**Methods:**

Using a dataset of 85,477 CCT images from 337 patients, we proposed a framework that consists of several processes that perform a combination of tasks including the selection of images with LA from all other images using a ResNet50 classification model, the segmentation of images with LA using a UNet image segmentation model, the assessment of the quality of the image segmentation task, the estimation of LAV, and quality control (QC) assessment.

**Results:**

Overall, the proposed LAV estimation framework achieved accuracies of 98% (precision, recall, and F1 score metrics) in the image classification task, 88.5% (mean dice score) in the image segmentation task, 82% (mean dice score) in the segmentation quality prediction task, and *R*^2^ (the coefficient of determination) value of 0.968 in the volume estimation task. It correctly identified 9 out of 10 poor LAV estimations from a total of 337 patients as poor-quality estimates.

**Conclusions:**

We proposed a generalizable framework that consists of DL models and computational methods for LAV estimation. The framework provides an efficient and robust strategy for QC assessment of the accuracy for DL-based image segmentation and volume estimation tasks, allowing high-throughput extraction of reproducible LAV measurements to be possible.

## Introduction

Cardiac computed tomography (CCT) is commonly used to evaluate pulmonary vein (PV) anatomy and left atrial (LA) appendage thrombus prior to catheter ablation (CA). These images can also be used for full volumetric LA measurement, as there is strong evidence of the association of LA volumes (LAV) with atrial fibrillation (AF) recurrence after CA (1, 2). Evidence also exists that suggests an association between LAV and successful PV isolation in CA of AF (3). The anatomic structure of LA is varied and complex, limiting precision of echocardiographic LAV measurements and making manual contouring and volume measurements of CCT time-consuming and user-dependent.

Recent popularity of the use of artificial intelligence (AI), a concept of developing and using computer algorithms with human-like intelligence to solve specific tasks, in healthcare is driven by the advances and accessibility to high-performance scalable computing equipment, prompting an expansion of research into new AI methods and algorithms. AI algorithms have the potential to interpret biomedical and healthcare data. These algorithms can be used to optimize care pathways, standardize clinical diagnosis and develop predictive models (4). The choice of the method, data quality and context associated with the way data is used are important for the success of an AI approach for a given problem. For example, deep learning (DL) algorithms, which are a set of techniques based on neural networks designed to achieve AI, are particularly suitable for medical imaging segmentation problems (5, 6). Medical image segmentation helps to quantify anatomical structures and produce quantities that are used to diagnose, monitor or prognosticate diseases. AI-based image segmentation tools are particularly useful in that they encourage standardization and consistency, reducing variability that characterizes manual human segmentation. They also permit high-throughput extraction of reproducible measurements that can be used for further analyses.

It is costly if image-derived variables that support clinical decisions in diagnosis and treatments are obtained from low quality or corrupted images, e.g., due to artifacts or noise introduced during image acquisition. Importantly, with the increasing use of DL models for medical image processing and analysis, it is pertinent that quality control (QC) mechanisms are in place to ensure cases where such models fail in deducing quantitative measures are flagged or identified. In clinical research, such failed cases can be processed manually or discarded altogether in subsequent statistical analysis. However, to the best of our knowledge, to date, QC has not been applied to LAV estimation obtained via DL approaches. Of course, LAV estimation machine learning (ML) methods have been proposed for some modalities (7–9) other than CT, but even these have only been developed with very limited amount of data (e.g., 56–58 subjects), meaning that, the generalizability of these methods have not been established. Importantly, in tasks like LAV estimation using DL in which image segmentation is only part of the process, the errors at the segmentation level can add up cumulatively in the computation of the volumes. QC assessment via visual inspection of each segmentation mask is time consuming and infeasible, particularly for large datasets. Thus, there is the need for a QC framework for DL-based LAV estimations that is generalizable to large datasets and can identify cases that are difficult for DL models to read or where the predictions of such models are inaccurate.

In this study, we present a novel fully automated framework for estimating LAV from CCT images. The framework consists of several processes that perform a combination of tasks including the selection of images with LA from all other images using a DL classification model, the segmentation of images with LA using a DL image segmentation model, the assessment of the quality of the image segmentation task, the estimation of LAV by integrating the interslice volumes computed using segmentation areas across neighboring slices and QC assessment. Existing automated DL methods only combine some of these essential processes and not all, making their practical utility and general adoption of such algorithms limited, particularly for large cohort studies. Using a dataset of 85,477 contrast-enhanced CCT images from 337 patients, we show that our approach provides an efficient and robust methodology for fully automated LAV estimation. The proposed approach is also equipped with a QC mechanism that allows LAV estimations to be flagged for rejection if it determines that the prediction is of poor quality. Thus, the framework allows high-throughput extraction of reproducible LAV measurements.

## Methods

**Figure 1:**
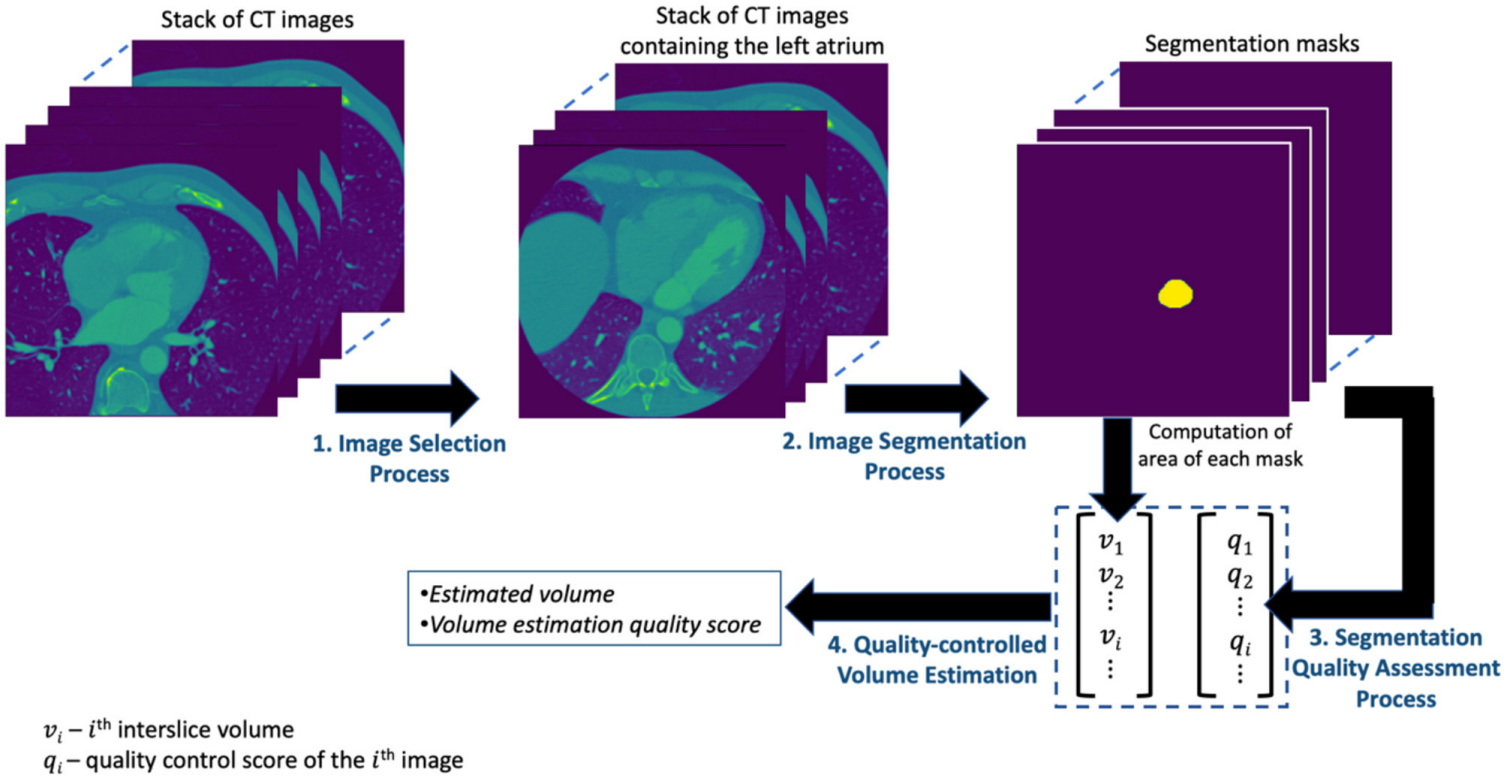
The overview of the proposed framework for estimating the volume of LA.

The presentations related to DL in this paper follow the relevant aspects of the recommendation of the Proposed Requirements for Cardiovascular Imaging-Related Machine Learning Evaluation (PRIME) guidance (10). The overview of the proposed framework for estimating the volume of LA is shown in Figure 1 and consists of four main processes. The image selection process performs an image classification task for selecting images containing the LA from the set of all CT images acquired for a given patient. The image segmentation process obtains the segmentation mask from the selected images of the preceding process. The segmentation quality assessment process quantifies the quality of the segmentation task while the quality-controlled volume estimation process computes the LAV and quantifies the accuracy of that computation. Each of these processes is described in detail in subsequent subsections.

### Data Acquisition and Analysis Tools

#### Study Population

The study included 337 patients who underwent catheter ablation for symptomatic, anti-arrhythmic medication-refractory atrial fibrillation. This retrospective observational study was performed based on analysis of CCT within a database from MedStar Georgetown University Hospital in Washington, D.C. All patients underwent CCT for pre-operative assessment and gave written informed consent. Images were de-identified prior to analysis. This study was approved by the Georgetown University Institutional Review Board (STUDY-0400, approved 7/20/2017). A total of 85,477 images for all the 337 patients were available for the study. This dataset and its subsets are used for all the experiments (analysis, modeling, and evaluation) in this paper. The baseline characteristics of the studied patients are given in Table 1.

**Table 1:** Baseline characteristics of the patients.

**Baseline characteristics**	**Total (*N* = 337)**
Age, years	63.2 (± 10.2)
Men, *n* (%)	223 (66)
BMI (kg/m^2^)	31.1 (± 7.0)
LAV (ml)	137.1 (± 46.4)
Paroxysmal AF, *n* (%)	219 (65)

*Values are numbers and percentage (%) of the variables (± standard deviation)*.

*AF, atrial fibrillation; BMI, body mass index; LAV, left atrial volume*.

#### CT Acquisition

Pre-operative CCT was acquired using a high resolution 256-slice scanner (Brilliance iCT; Royal Philips; Amsterdam, Netherlands) with 0.625 mm detector collimation. Images were acquired using prospectively ECG-triggered CCT scanning at 40% of the cardiac cycle, capturing the chest from carina to diaphragm during the angiodynamic administration of contrast medium. The contrast protocol included administration of 60 mL iohexol (Omnipaque; GE Healthcare; Chicago, Illinois) intravenously at a rate of 5 mL/s with a kV range between 80 and 120.

#### Manual Segmentation of LA as Ground Truth Dataset

CCT scans were analyzed using the semi-automated post-processing program 3D Slicer (Version 4.11.0, http://www.slicer.org). The LAV was calculated based on manual segmentation using axial views: the LA was encircled on each 2D slice of the CCT scan superiorly to inferiorly by a reader with several years of experience. Care was taken to exclude the left atrial appendage and pulmonary veins. 3D Slicer calculates the area of each corresponding LA section and estimates the LAV based on the distance between adjacent planes.

#### Machine Learning Software Tools

All experiments were conducted in Python programming language with TensorFlow 2.0 Python API machine learning framework, available from https://www.tensorflow.org/ on a Nvidia Tesla M40 machine. The source code for the experiments can be found on the following GitHub repository: https://github.com/mabdulkareem/lav_volume_with_qc.

### Image Classification Model

We consider three DL architectures that can be used for the image classification task of selecting images containing the LA from the set of all CT images acquired for a given patient. These architectures are based on the VGG (VGG16 and VGG19) (11) and the ResNet (ResNet50) (12) architectures. The configuration of the VGG16 and VGG19 architectures are given in Table 2. For each of these two VGG architectures, the pooling layer after the last convolutional layer of the network is followed by a global average pooling operation and then a dense layer of 1 unit (neuron) with the sigmoid nonlinear activation function applied. The sigmoid function is chosen given that we are attempting to solve a binary (two-class) classification problem, that is, determine the presence or the absence of LA in a given CCT image. Each of the convolutional layers uses rectified linear unit (ReLU) as its activation function.

**Table 2:**
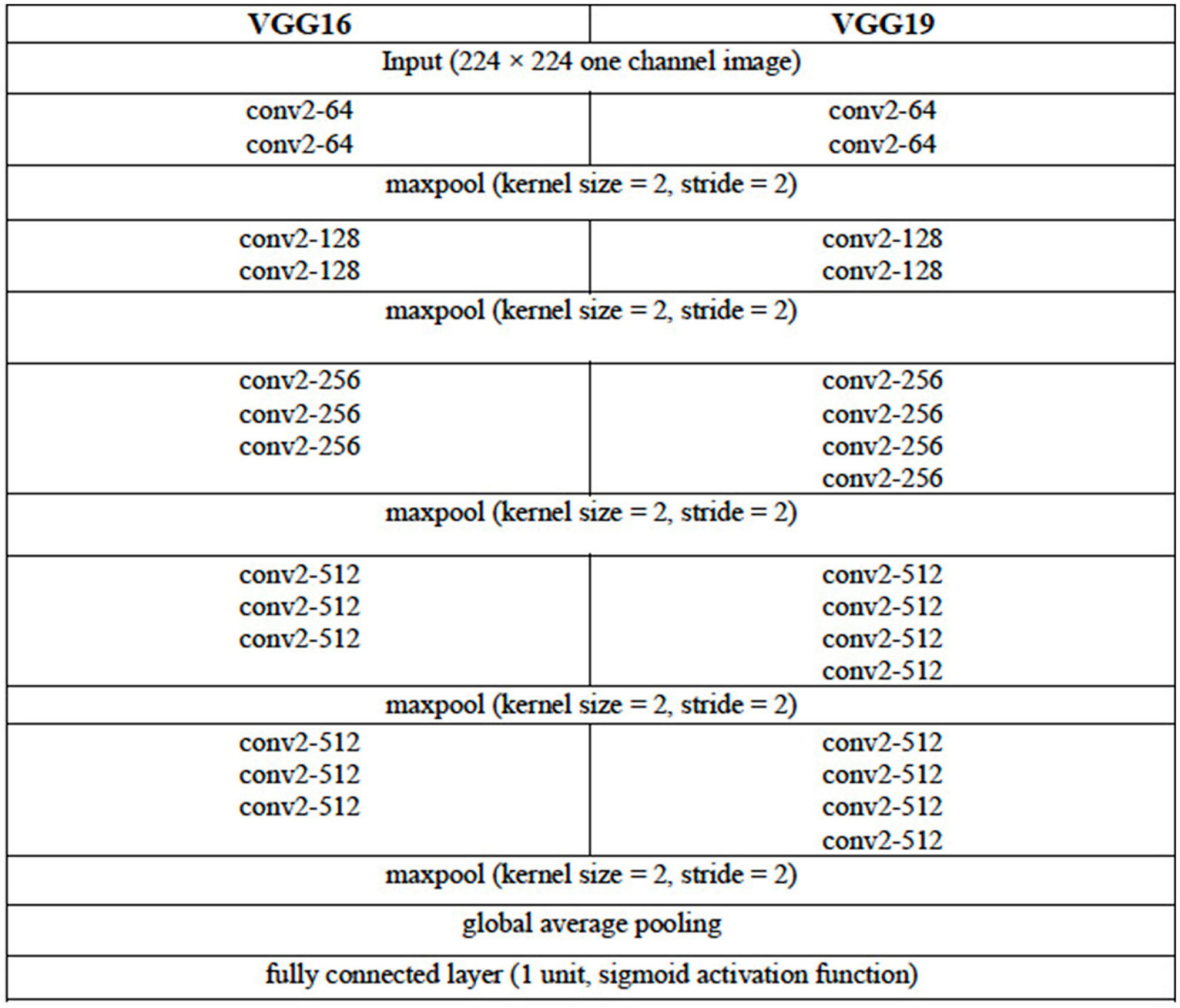
Configuration of the VGG16 and VGG19 DL Architectures.

*The convolutional layer parameters are denoted as “conv2-(number of filters),” where “conv2” denotes 2D convolution operation and the height and width of the 2D convolution window is 2 × 2. For brevity, the ReLU activation function is not shown*.

The ResNet50 architecture consists of five stages. The first stage consists of one convolutional layer and each of the remaining four stages consists of several blocks of convolutional layers with skip connections. These blocks are referred to as identity blocks if they contain no convolutional layer in the skip connection path, otherwise, they are called as convolutional block. Similar to the VGG16 and VGG19 architectures in this work, the last convolutional layer of the ResNet50 architecture is followed by a global average pooling operation and then a dense layer of 1 unit (neuron) with the sigmoid nonlinear activation function applied and the outputs of the model yields a binary prediction (i.e., an image is with/without LA). The configuration of the ResNet50 architecture is given in Table 3. The number of parameters of the three classification models are given in Table 4.

**Table 3:**
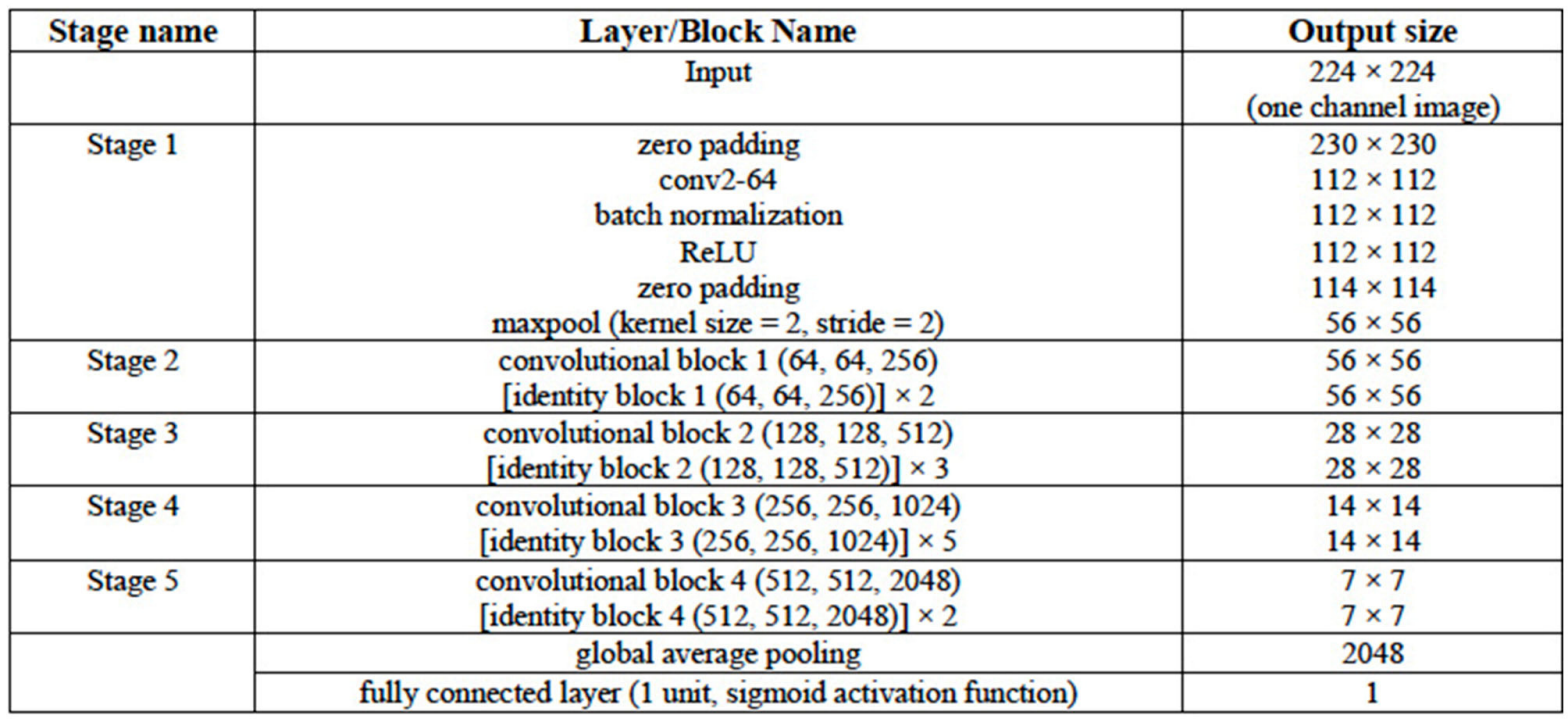
Configuration of the ResNet50 DL Architecture.

*The convolutional layer parameters are denoted as “conv2-(number of filters),” where “conv2” denotes 2D convolution operation and the height and width of the 2D convolution window is 2 × 2. Each of the convolutional or identity blocks is followed by the number of filters n_i_ of its three 2D convolutional operations as in “convolutional block x (n_1_, n_2_, and n_3_).” Two or more identity blocks stacked together are denoted as “[identity block x (n_1_, n_2_, and n_3_)] × k,” where k denotes the number of blocks stacked together*.

**Table 4:** Number of parameters of the DL Architectures.

	**VGG16**	**VGG19**	**ResNet50**
Number of trainable parameters	14,714,049	20,023,745	23,530,369
Total number of parameters	14,714,049	20,023,745	23,583,489

For the training of the VGG16, VGG19, and ResNet50 classification models, the input CCT images were resized to 224 × 224. For generalizability of the architectures, we rotated the images up to ±10° and their intensities normalized as part of the on-the-fly data augmentation (which ensures a different set of images is used after each complete training epoch). The parameters (weights) of the models were randomly initialized and training proceeded for 60 complete epochs using a batch size of 128 images. The binary cross-entropy function was used as the loss function. The optimization method used was the Root Mean Squared Propagation (RMSProp) optimizer, with an initial learning rate of 1e-4, decreasing as shown in Table 5. If 24 epochs elapsed with no decrease in the loss function, training was set to cease and the weights from the best epoch (i.e., one that yielded the lowest loss) is restored as the model's weights.

**Table 5:** Learning rate adopted for training the image classification models with *lr* = 1e-4.

**Epoch**	**1–10**	**11–15**	**16–20**	**21–25**	**26–30**	**31–35**	**36–40**	**41–45**	**46–50**	**51–60**
Learning rate	*lr*	0.5 × *lr*	0.2 × *lr*	0.1 × *lr*	0.05 × *lr*	0.02 × *lr*	0.01 × *lr*	0.005 × *lr*	0.002 × *lr*	0.001 × *lr*

The CT image datasets of 150 patients (Dataset 1) of which with 15,961 images contain LA were used to train, validate and test the three models. Dataset 1 consists of 42,296 CT images in total. Of these, 15% of these images were selected and divided into two equal sets representing the validation and test sets. Specifically, the number of the training, validation and test (evaluation) images are 35,952, 3,172, and 3,172, respectively.

Of the 35,952 images that constitute the training set, the number of images with LA present and absent are 13,586 and 22,366, respectively. In order to address this data imbalance, we used weighted loss function. Let {(*x*_1_, *y*_1_), (*x*_2_, *y*_2_), …, (*x*_*n*_, *y*_*n*_), …, (*x*_*N*_, *y*_*N*_)} denote a training set of *N* samples where *x* is the two-dimensional input image and *y* ∈ {0, 1}^*C*^ denote a binary one-hot encoded label with *C*=2 in our case, then the weighted loss function is defined as:


(1) $$\begin{array}{l} E_{w}\left(\theta \right)=-\frac{1}{N}[\lambda_{0}\overset{N}{\underset{n=1}{\sum }}{𝕋}_{0}\left(x_{n}\right){\;y}_{n}\log\left({ŷ}_{n}\left(x_{n},\;\theta \right)\right)+\\ \;\lambda_{1}\overset{N}{\underset{n=1}{\sum }}{𝕋}_{1}\left(x_{n}\right)\;y_{n}\log\left({ŷ}_{n}\left(x_{n},\;\theta \right)\right)] \end{array}$$

where θ denotes the trainable parameters of a model; ŷ_*n*_(*x*_*n*_, θ) is the posterior probability obtained after the application of sigmoid activation function on the output layer of the model; 𝕋_0_(*x*_*n*_) and 𝕋_1_(*x*_*n*_) are functions that indicate whether image *x*_*i*_ belongs to class 0 or class 1, respectively (in our case, whether LA is absent or present in image *x*_*i*_, respectively); and λ_0_ and λ_1_ are weights that penalize the loss function for false negatives errors (*x*_*i*_ that belongs to class 0 is misclassified as belonging to class 1) and false positive errors (*x*_*i*_ that belongs to class 1 is misclassified as belonging to class 0), respectively. The weights, λ_0_and λ_1_, can be computed using the following formula:


(2) $$\begin{array}{l} \lambda_{i}=\frac{1}{k_{i}}\cdot \frac{N}{C} \end{array}$$

where *k*_*i*_ is the number of images in class *i*. In our case, *N* = 35952; class 0 and class 1 are subgroups indicating the collection of samples where LA is absent and present, respectively; then, $\lambda_{0\;}=\left(\frac{1}{22366}\right)\times \left(\frac{35952}{2}\right)=0.8037$ and $\lambda_{1\;}=\left(\frac{1}{13586}\right)\times \left(\frac{35952}{2}\right)=1.3231$. In other words, the images contain LA (class 1) are weighted as being more valuable than those without LA (class 0) since the original Dataset 1 is skewed (i.e., images in class 0 are more than those in class 1).

### Image Segmentation Model

The configuration of the UNet DL architecture (13) that is used for the image segmentation task is given in Table 6. The UNet architecture includes batch normalization following every convolutional layer to enhance robustness of the model [i.e., reduce sensitivity to initial parameters and learning rates (14)]. Also, “dropout” operation (15), dropping out 30% hidden neurons, is performed on the first six consecutive up-sampling convolutional layers of the architecture to avoid problems associated with model overfitting. The output of the model yields an image segmentation mask with the background pixels labeled 0 and the foreground pixels (region of the LA) labeled l. The total number of parameters of the model is 29,812,034 out of which 29,800,514 parameters are trainable.

**Table 6:**
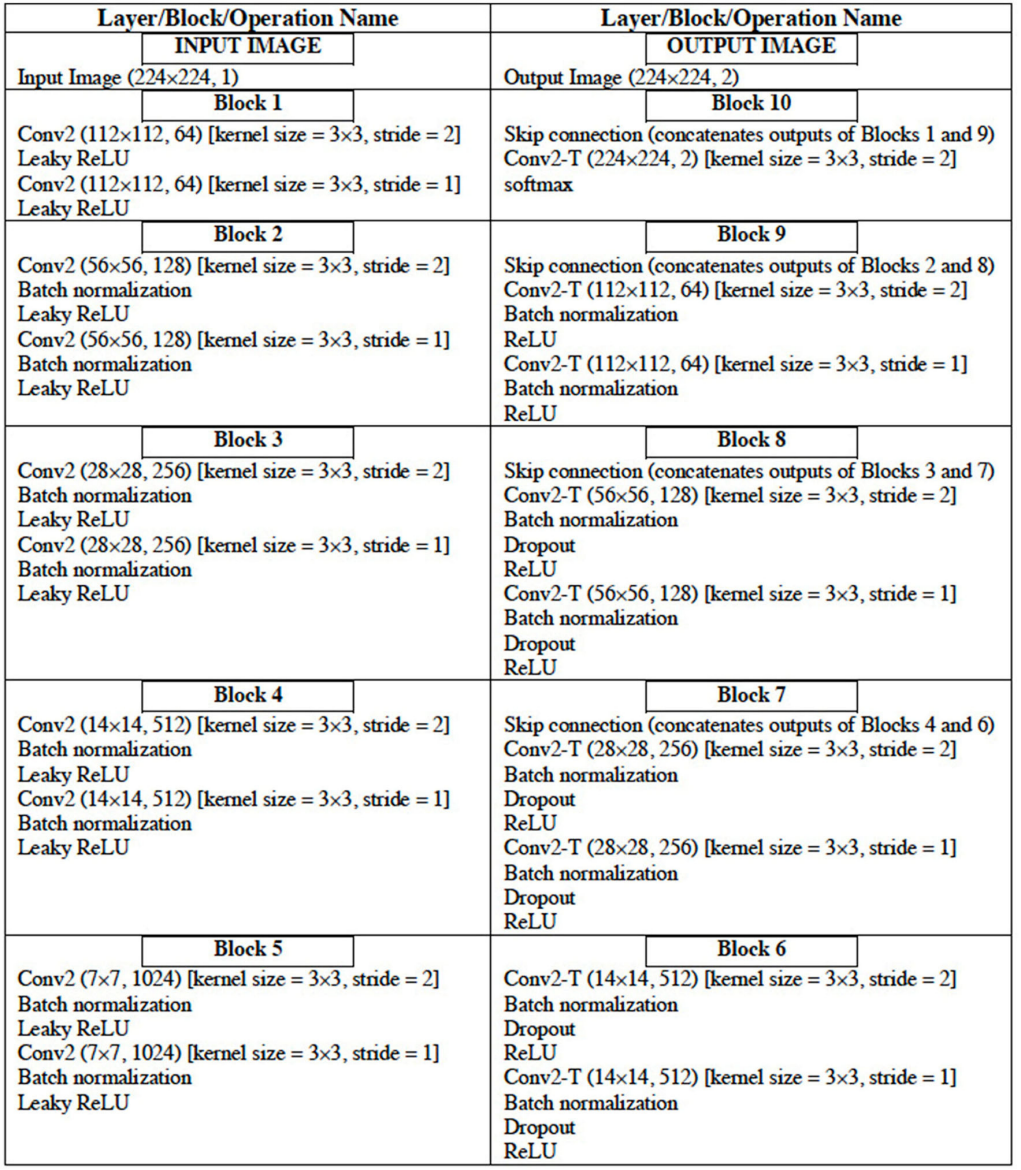
Configuration of the UNet DL Architecture.

*The convolutional layer parameters are denoted as “conv2 (dimension of output, number of filters)” where “conv2” denotes 2D convolution operation. “conv2-T (dimension of output, number of filters)” denotes 2D transpose convolution layer. The conv2 and conv2-T operations have ‘same' padding (i.e., output and input of the operation have the same height and width)*.

For the training of the UNet model for image segmentation, the input CCT images were resized to 224 × 224. The images were rotated up to ±30° and their intensities normalized as part of the on-the-fly data augmentation. The parameters of the models were randomly initialized and training proceeded for 60 complete epochs using a batch size of 64 images. The sparse categorical cross-entropy function was used as the loss function. The optimization method used was the Adam optimizer, with an initial learning rate of 1e-4. If 24 epochs elapsed with no decrease in the loss function, training was set to cease and the weights from the best epoch is restored as the model's weights. The optimization method used was the Adam optimizer, with an initial learning rate of 1e-4, decreasing as shown in Table 7.

**Table 7:** Learning rate adopted for training the UNet image segmentation model with *lr* = 1e-4.

**Epoch**	**1–10**	**11–20**	**21–30**	**31–35**	**36–40**	**41–45**	**46–50**	**51–60**
Learning rate	*lr*	0.5 × *lr*	0.2 × *lr*	0.1 × *lr*	0.05 × *lr*	0.02 × *lr*	0.01 × *lr*	0.005 × *lr*

For the training set {(*x*_1_, *y*_1_), (*x*_2_, *y*_2_), …, ( x_n , y_n ), …, (*x*_*N*_, *y*_*N*_)} of *N* samples where *x* is the two-dimensional input image and *y* denote the two-dimensional segmentation mask, then the sparse categorical cross-entropy loss function is defined as:


(3) $$\begin{array}{l} E_{w}\left(\theta \right)=-\frac{1}{N}\left[\overset{N}{\underset{n=1}{\sum }}{\;y}_{n}\log\left({ŷ}_{n}\left(x_{n},\;\theta \right)\right)\right] \end{array}$$

where θ denotes the trainable parameters of a model and ŷ_*n*_(*x*_*n*_, θ) is the posterior probability obtained after the application of “softmax” activation function on the output layer of the model.

The CT image datasets of 150 patients in Dataset 1 with LA present (15,961) were used to train, validate and test the UNet models for the segmentation of LA. Of these, 15% of these images were selected and divided into two equal sets representing the validation and test sets. The training, validation and test images are 13,567, 1,197, and 1,197, respectively.

### Image Segmentation Quality Assessment

One of the methods that have been proposed to address QC for AI-based segmentation tasks is the so-called Reverse Classification Accuracy (RCA) method (16, 17). The RCA algorithm attempts to predict the dice similarity coefficient (DSC), or dice score, using the segmentation mask in the absence of the ground truth. DSC is a measure of similarity between the label and predicted segmentation masks. Given two sets (two images in this case) *A* and *B*, DSC score can be expressed as follows:


(4) $$\begin{array}{l} DSC=\;\frac{2|A\cap B|}{\left(\left|A|+|B\right|\right)} \end{array}$$

where |*A*| and |*B*| represent the cardinalities of set *A* and *B* (i.e., the number of elements in each set), respectively. The DSC, which has a range of [0,1], is a useful summary measure of spatial overlap that can be applied to quantify the accuracy in image segmentation tasks. Rather than using one test image to validate a segmentation model, it can be used as a statistical validation metric by computing the DSC of several images from a segmentation model and evaluating the mean DSC, allowing reproducibility and comparison of the model with other models. For the purpose of this study, we will consider the segmentation model's performance as good if the mean DSC is >0.8 on evaluation dataset.

The RCA algorithm consists of 3 main steps: *Step 1* involves the image registration (rigid registration) of some *N* number of (moving) images against the *k*^*th*^ (fixed) image to obtain a set of *N* transformation matrices; *Step 2* involves using the transformation matrices from *Step 1* to warp the segmentation masks of the *N* images—thereby obtaining a set of *N* warped segmentation masks; and *Step 3* involves the prediction of the dice score. Thus, for a given mask, say mask *m*, from the output of the DL segmentation model, a set of warped masks are computed using the transformation matrices obtained in *Step 1*; these warped masks of mask *m* are then compared with the all warped segmentation masks obtained in *Step 2* as ground truth in order to obtain the set of dice scores. The maximum dice score in the set of dice scores is assumed to be the predicted dice score—which is often an estimate that is good.

In our segmentation quality assessment approach, we modified the RCA method to improve its prediction accuracy—we call this the modified RCA (mRCA) method. Specifically, rather than using one fixed image in *Step 1*, we used several fixed images—thereby, building an atlas-like knowledge base. From *M* = 704 images from *p* = 5 patients (with patients selected at random) containing LA and their segmentation masks, starting from the first image of these stacked layers of images, we selected images at 25 slice intervals—a set of *K* = 29 images in total—for obtaining the transformation matrices and warped segmentation masks. In particular, the transformation matrices {*T*_1_, *T*_2_, …, *T*_*N*_} and warped segmentation masks {*w*_1_, *w*_2_, …, *w*_*N*_} were computed by selecting each of these 29 images as the *k*^*th*^ image used as the fixed image in the rigid registration process of *Step 1* and the remaining 28 images as the moving images—iterating through all the 29 images in the set. In total, we have *N* = 812 (i.e., 29 × 28) transformation matrix and warped segmentation mask pairs in *Step 2*. Thus, in *Step 3*, in order to predict the dice score of mask *m*, the 812 transformation matrices are used to warp *m* and each of these warped masks is compared with its corresponding warped segmentation mask to obtain a list of 812 dice scores *D* = {*d*_1_, *d*_2_, …, *d*_*N*_}. The maximum dice score in this list, that is max(*D*), is the predicted dice score for mask *m*.

**Figure 2:**
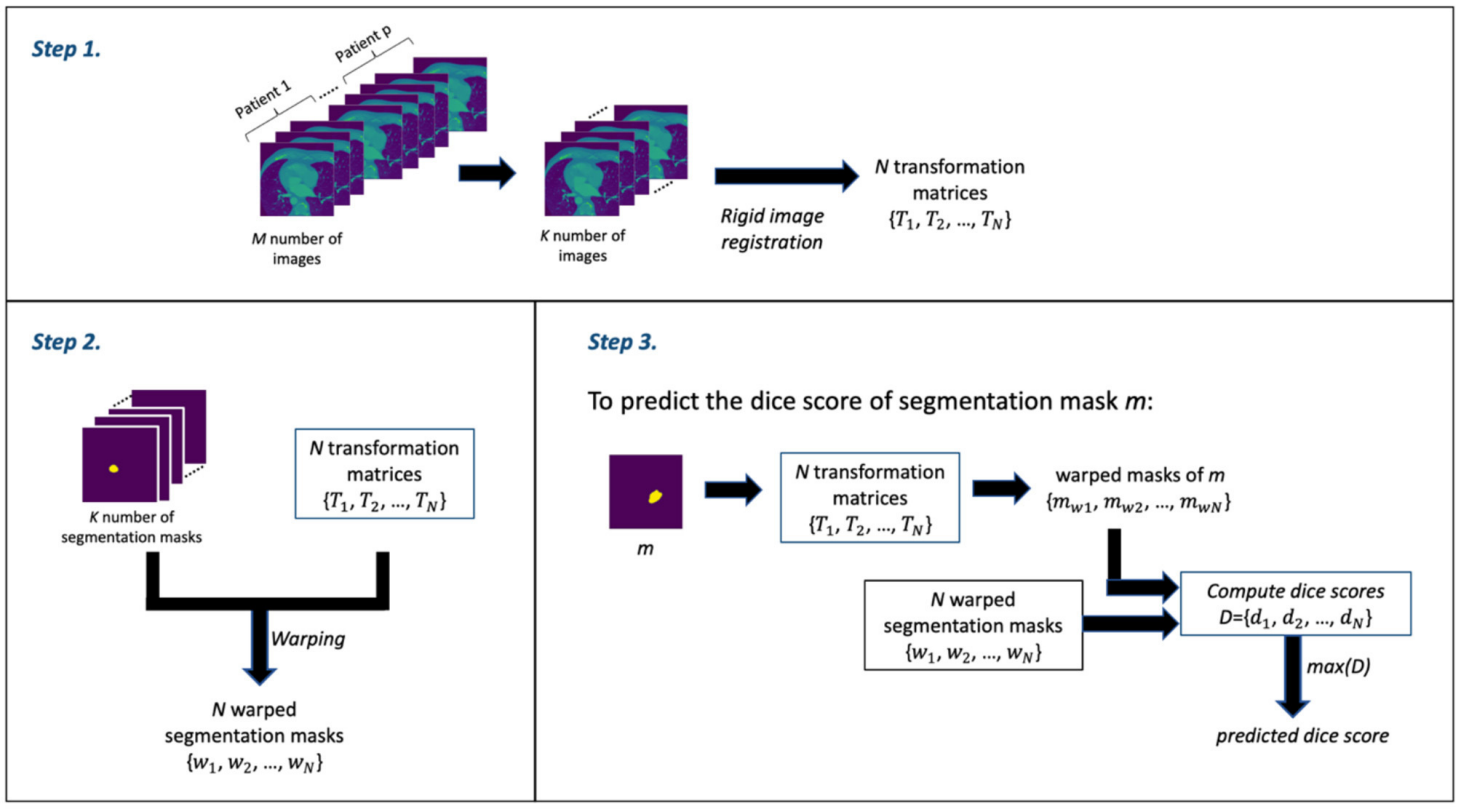
The three steps of the Modified Reverse Classification Accuracy (mRCA) method.

The *M* = 704 images from *p* = 5 patients is a subset of the entirety of images (1,315). This subset is the complete selection of images in which LA is present. We find using several images across several patients and across several slices in the mRCA method built using 29 images superior in dice score prediction accuracy than using 100 images built with the RCA method in the original paper. Figure 2 shows a schematic description of the mRCA method.

### Volume Estimation and Quality Control

The estimation of LAV involves the summation of the interslice volumes where each interslice volume is estimated by computing the approximate volume between two consecutive slices using the following formula:


(5) $$\begin{array}{l} V=\;\overset{N}{\underset{i}{\sum }}v_{i} \end{array}$$

where *V* is the estimated LAV, *N* is the number of slices, and *v*_*i*_ is the *i*th interslice volume and is defined as follows:


(6) $$\begin{array}{l} v_{i}=\frac{A_{i}\;+\;A_{i+1}}{2}z_{k_{i}} \end{array}$$

where *z*_*k*_*i*__ is the distance between two consecutive slices in the *z* direction and is given by $z_{k_{i}}=z_{i}\hat{k}$, where $\hat{k}$ is the unit vector in the *z* direction and *z*_*i*_ is the perpendicular distance between two consecutive parallel slices; *A*_*i*_ and *A*_*i*+1_ are the areas of the two consecutive slices. That is, *v*_*i*_ is the arithmetic mean of the areas of two consecutive slices multiplied by the distance between them in the direction of the *z*-axis. Also, *A*_*i*_ can be obtained as follows:


(7) $$\begin{array}{l} A_{i}=n_{i}s_{x}s_{y} \end{array}$$

where *n*_*i*_ is the number of pixels that constitutes the LA area on the *i*th slice; *s*_*x*_ and *s*_*y*_ are the pixel dimensions in the *x* and *y* directions, respectively; and $A_{i}=n_{i}s^{2}$ where *s* = *s*_*x*_ = *s*_*y*_.

In order to assess the quality of volume estimation, it is tempting to try to check the quality of the segmentation mask of each slice using the mRCA technique described in the preceding section but computational time increases significantly in doing so. Because neighboring slices are often similar, we propose to assess the quality of slices at specific slice intervals. For example, in order to assess the quality of LAV estimation and given that neighboring slices are often similar, we propose to assess the quality of slice at 8 slice intervals using the following two steps:

*Step 1* Estimate the segmentation quality by computing the set of dice scores, 𝔻, at 8 slice intervals using mRCA method; that is, 𝔻 = {*d*_1_, *d*_9_, *d*_17_, … }.

*Step 2* Compute the following:

- *QC Score Mean*, *q*_*mean*_: the arithmetic mean of 𝔻.
- *QC Score Percentage*, *q*_*perc*_: the percentage of entries of 𝔻 with value of *d*_*i*_ > 0.7.
- *QC Score Percentage Number*, *q*_*no*_: the number of entries of 𝔻 with value of *d*_*i*_ > 0.7.

The scores *q*_*mean*_, *q*_*perc*_, and *q*_*no*_ can then be used as a QC mechanism by the imposition certain conditions on them in order to determine whether to accept or reject an estimated LAV computation.

In addition, in order to compare the predictions of the RCA and mRCA methods against the actual DSC scores, the mean absolute error (MAE) and mean squared error (MSE) may be used. The MAE and MSE are defined as follows:


(8) $$\begin{array}{l} MAE=\frac{1}{n}\overset{n}{\underset{i=1}{\sum }}|e_{i}| \end{array}$$

(9) $$\begin{array}{l} MSE=\frac{1}{n}\overset{n}{\underset{i=1}{\sum }}{e_{i}}^{2} \end{array}$$

where error *e*_*i*_ = *y*_*i*_ − ŷ_*i*_, and *y*_*i*_ and ŷ_*i*_ represent the label and predicted values, respectively.

## Results

### Image Classification Model

**Figure 3:**
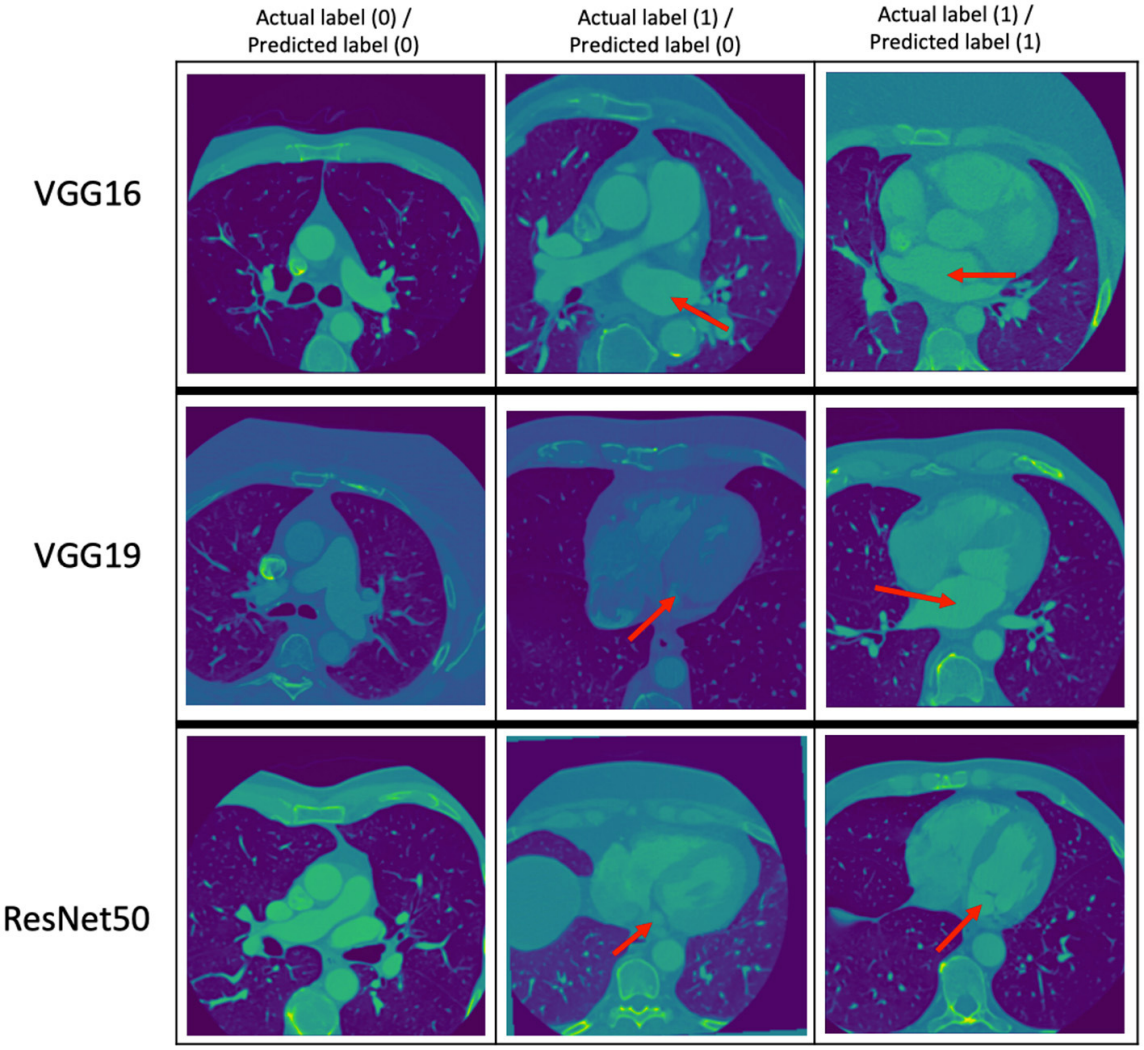
Some examples of the prediction of the classification models. The top, middle, and bottom rows correspond to the predictions of the VGG16, VGG19, and ResNet50 models, respectively. The red arrows point to the LA. The middle column shows images with LA that the classification models got wrong.

The performance metrics (precision, recall, and F_1_ score) of the VGG16, VGG19, and ResNet50 trained models on the same evaluation dataset are given in Table 8 while the confusion matrices are given in Table 9. Figure 3 provides some examples of the predictions of the classification models. The results show that ResNet50 model outperforms both VGG16 and VGG19 models on all the metrics. As such, we selected ResNet50 model as the basic classification model for our framework in the rest of this paper.

**Table 8:** Performance metrics for the classification models using the evaluation dataset (*N* = 3,172).

**Model**	**VGG16**	**VGG19**	**ResNet50**
Precision	0.9554	0.9605	0.9754
Recall	0.9342	0.9419	0.9779
F1 score	0.9447	0.9511	0.9766

**Table 9:** Confusion matrices for the classification models using the evaluation dataset (*N* = 3,172) with class 0 (absence of LA in an image) and class 1 (presence of LA in an image).

**Model**	**VGG16**		**VGG19**		**ResNet50**	
	***Predicted label (0)***	***Predicted label (1)***	***Predicted label (0)***	***Predicted label (1)***	***Predicted label (0)***	***Predicted label (1)***
*Actual Label (0)*	1,951	51	1,939	46	1,969	29
*Actual Label (1)*	77	1,093	69	1,118	26	1,148

*LA, left atrium*.

### Image Segmentation Model With Quality Assessment

**Figure 4:**
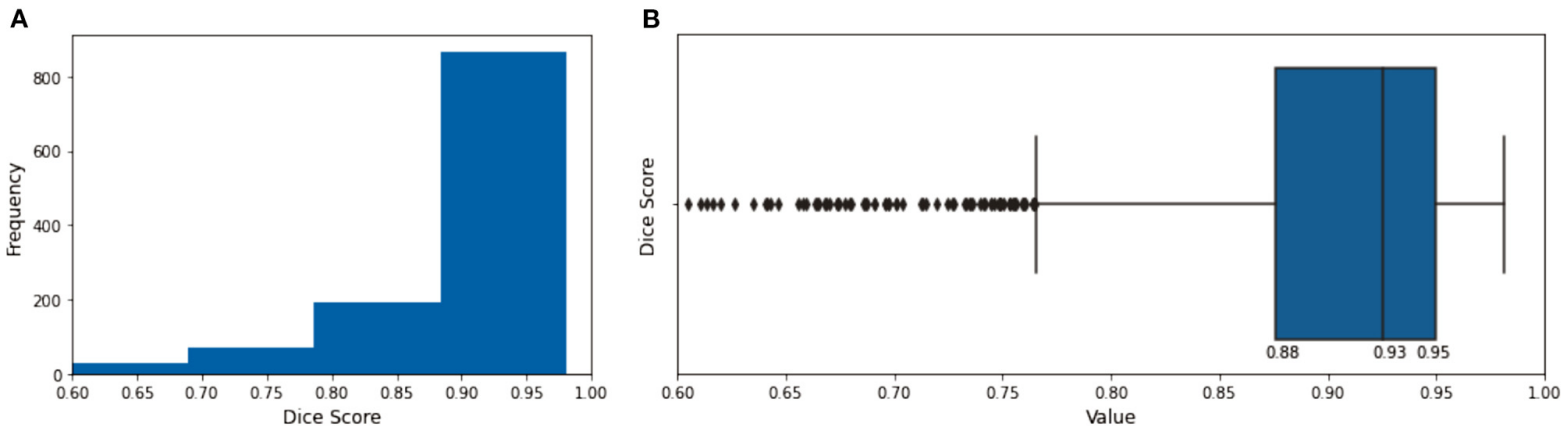
The histogram **(A)** and the boxplot **(B)** of the dice score of the evaluation dataset. The 25, 50, and 75% percentiles are 0.88, 0.93, and 0.95, respectively.

**Figure 5:**
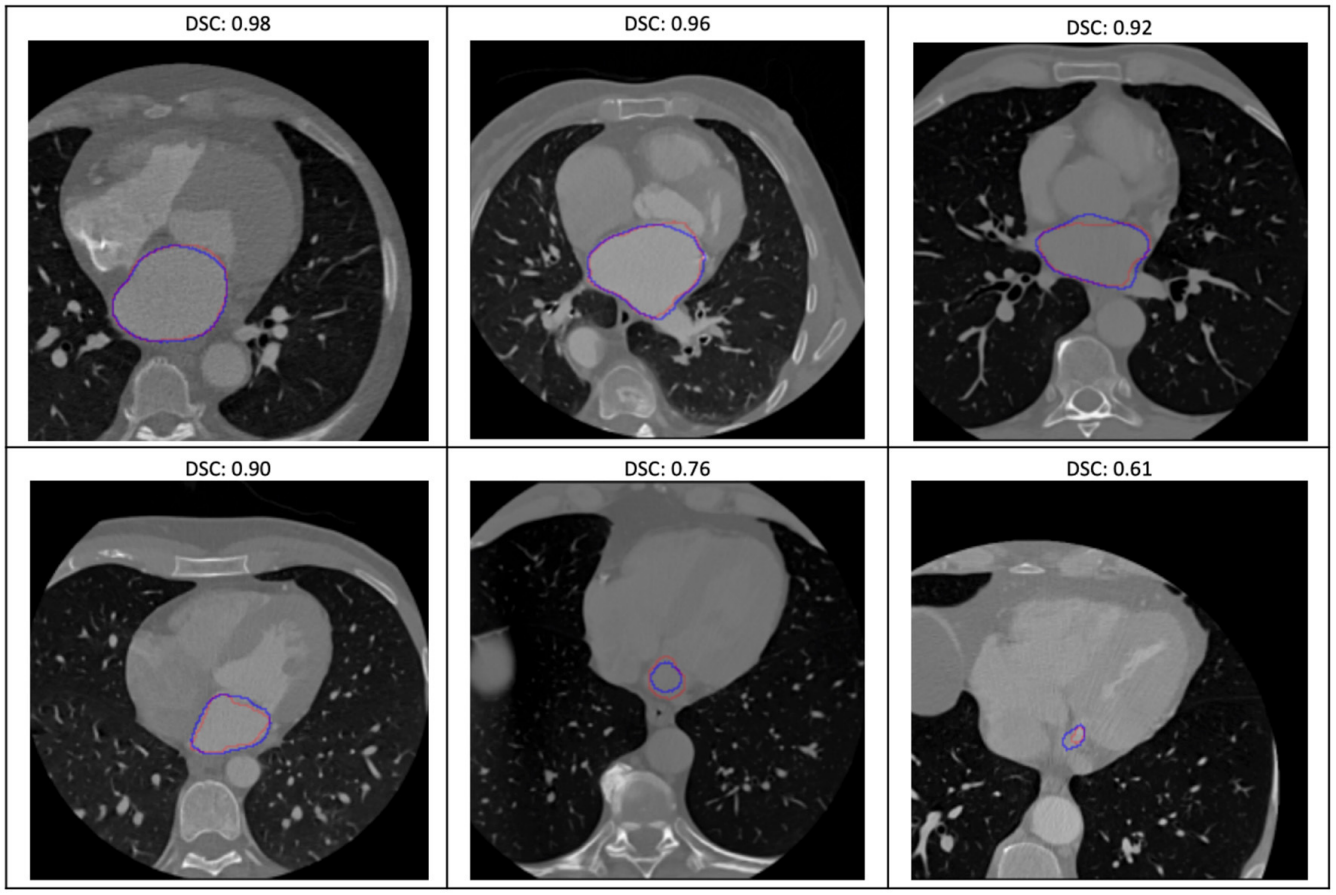
Some examples of the variable prediction of the segmentation model (the red and blue contours represent the label and prediction and the corresponding dice scores are shown on the top of each image).

The evaluation of the predicted results of the UNet model was performed using the DSC score, a measure of similarity between the label and predicted segmentation masks, as the performance metric. For the 1,197 evaluation set, the mean DSC is 0.885 (± 0.12 standard deviation; 25, 50, and 75% percentiles are 0.88, 0.93, and 0.95, respectively; the maximum is 0.98). The distribution of the dice score is given in the histogram and boxplot of Figure 4. Figure 5 displays some examples of the predictions of the segmentation model. Overall, the performance of the segmentation model was good (mean DSC > 0.8).

**Figure 6:**
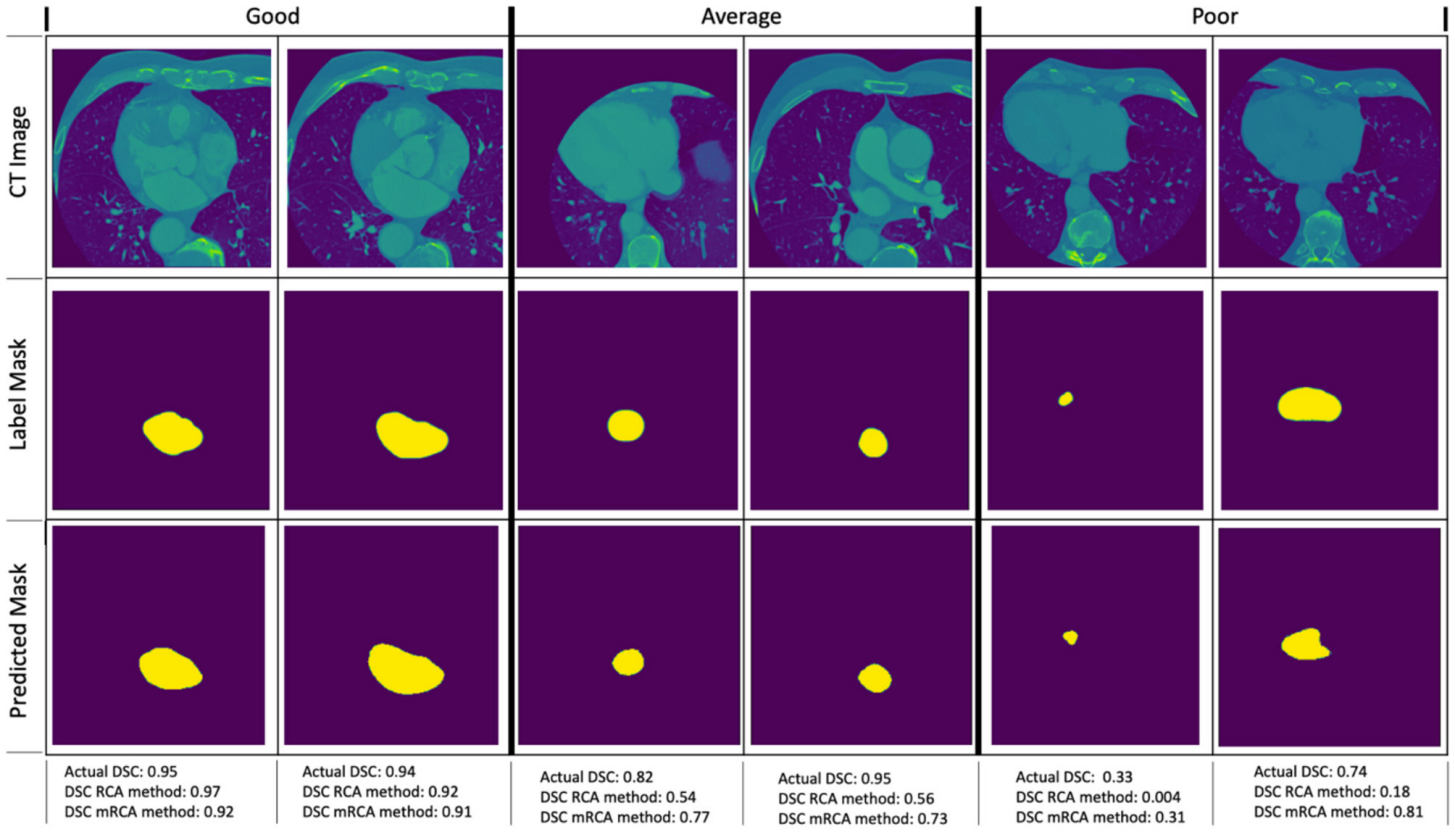
Examples of image quality assessment comparing RCA and mRCA methods. The “Good” (η ∈ [0 0.2]), “Average” (η ∈ [0.2 0.5]), and “Poor” (η ∈ [0.5 1.0]) refer to the quality of the predictions of the RCA method when compared to the actual DSC scores in terms of the relative error (not shown on the figure), where relative error η = |(*DSC*_*actual*_ − *DSC*_*predicted*_)/*DSC*_*actual*_|).

Figure 6 shows some examples of image segmentation quality assessment. For an evaluation dataset of 2,000 test images randomly selected from 15,961 images with LA of Dataset 1, Table 10 shows the mean dice scores computed for the actual DSC and the predicted DSC with RCA and mRCA methods. The table also shows the MAE and MSE values comparing the predictions of these methods against the actual DSC scores. These metrics indicate that mRCA has smaller errors than the RCA method. The image classification process, the image segmentation process and the prediction of the dice score for an image takes 805 ms with RCA method and 4,385 ms with mRCA method.

**Table 10:** Mean of actual DSC and predicted DSC for RCA and mRCA methods with MAE and MSE results for 2,000 images.

	**Actual DSC**	**DSC with RCA**	**DSC with mRCA**
Mean DSC	0.88	0.52	0.82
MAE	–	0.3634	0.0899
MSE	–	0.1739	0.0231

*DSC, dice similarity coefficient; mRCA, modified reverse classification accuracy; MAE, mean absolute error; MSE, mean squared error; RCA, reverse classification accuracy*.

### Volume Estimation With Quality Assessment

**Figure 7:**
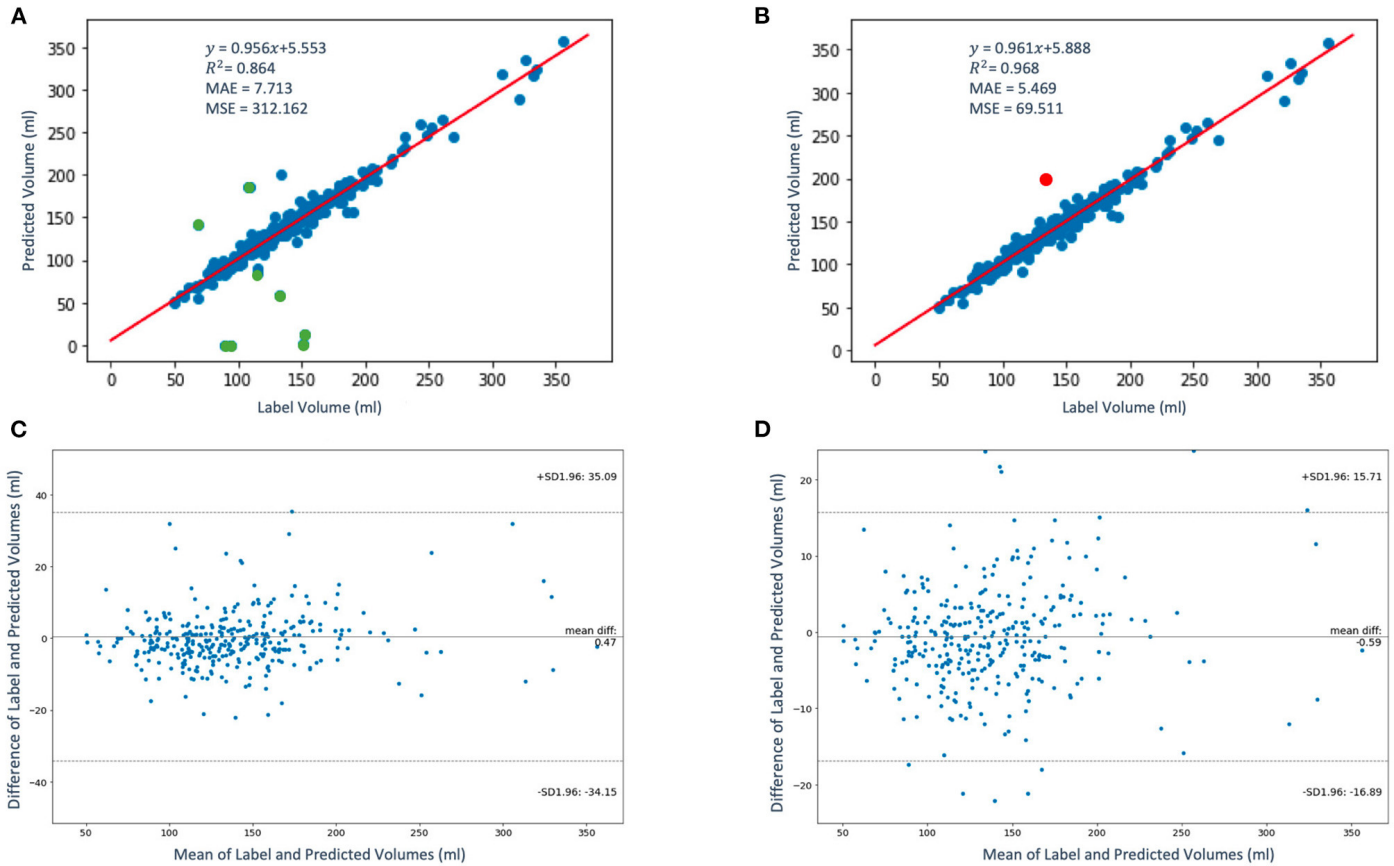
The regression **(A,B)** and the Bland-Altman **(C,D)** plots of the label volumes vs. the predicted volumes using the proposed framework. The data points are shown in blue and green in panel **(A)**; the green data points (9 out of 337) in the figure have been flagged by the QC condition as poor LAV estimates therefore eliminated in panel **(B)**. The red data point in panel **(B)** indicates one poor volume estimate (out of a total of 10) was missed by the imposed condition. In panels **(C,D)**, the upper and lower dashed horizontal lines represent the confidence interval at 95%.

**Figure 8:**
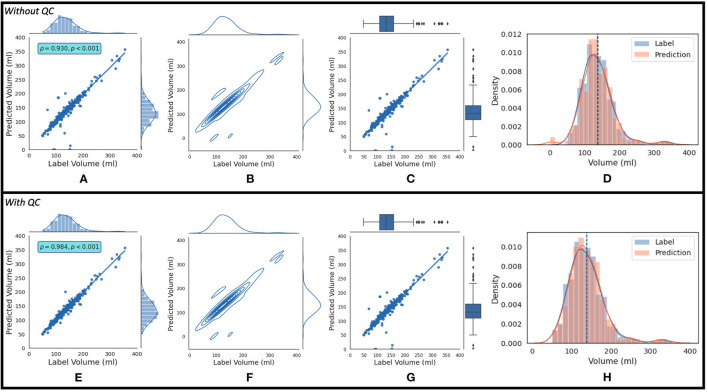
The top row shows **(A)** the regression plot and **(B)** the kernel density estimate plot along the two variables (label volume and predicted volume) marginal distributions. It also shows **(C)** the regression plot along the two variables' boxplots and **(D)** the kernel density estimate and histogram plots of the variables with the dashed vertical lines representing the arithmetic mean of the distributions. The symbols ρ and *p* represent the p-value and Pearson correlation coefficient, respectively. The plots **(A–D)** correspond to the label volumes vs. the predicted volumes without quality control (QC) and the plots **(E–H)** correspond to these variables using the proposed QC framework.

The regression and the Bland-Altman plots of the label volumes vs. the predicted volumes computed for the study population of 337 patients using the proposed framework are shown in Figure 7. Figure 7A shows the regression plot with volume computed using Equations (5–7) before QC assessment was implemented. In our case, *s*_*x*_ = *s*_*y*_ and *z*_*k*_*i*__ is approximately between 2.2 and 4.5 mm. Thus, without QC, the mean volume of the population is 137.38 ± 45.58 ml. In order to determine whether an LAV estimate is “poor” quality for a given patient using the scores *q*_*mean*_, *q*_*perc*_, and *q*_*no*_, we express the condition to be satisfied as follows:


$$\begin{array}{c} \left(\left(q_{mean}<0.6\right)\;AND\;\left(q_{perc}<60\right)\;AND\;\left(q_{no}<4\right)\right)\;AND\;\\ \left(\left(q_{mean}<0.65\right)\;OR\;\left(q_{perc}<20\right)\;OR\;\left(q_{no}<\;3\right)\right) \end{array}$$

where *AND* and *OR* are logical operators. If this condition is not satisfied, the estimated LAV value is of “good” quality. Figure 7B shows the regression plot after the imposition of this condition. With QC, the mean volume of the population is 138.38 ± 45.58 ml. The degree of linear correlation between the label and predicted quality-controlled volumes, *R*^2^, is 0.864 for the study population of 337 patients without QC. With QC, *R*^2^ is 0.968. Bland-Altman plots without and with QC are shown in Figures 7C and 7D, respectively. Also, 95% of the difference between the label and predicted volume values will be between −34.15 and +35.09 of the mean volume difference value of 0.47 for Figure 7C, and between −16.89 and +15.71 of the mean volume difference value of −0.59 for Figure 7D. In addition, Figure 8 shows (a) the regression plot and (b) the kernel density estimate plot along the label and predicted LAV marginal distributions. It also shows (c) the regression plot along the two variables' boxplots and (d) the kernel density and histogram plots of the variables with the dashed vertical lines representing the arithmetic mean of the distributions. The plots of Figures 8A–D correspond to the label volumes vs. the predicted volumes without quality control (QC) and the plots (Figures 8E–H) correspond to these variables using the proposed QC framework. The estimation of LAV with and without QC took ~74.25 s per patient and 23.32 s per patient, respectively. Table 11 shows five examples of the predicted volumes using the proposed framework.

**Table 11:** Examples of label and predicted LAV with QC scores for 5 selected patients with No. 1–4 being of good quality and No. 5 flagged as being of poor quality.

**No**.	**Label volume**	**Predicted volume**	**q_mean_**	**q_perc(%)_**	**q_no_**
1	146.6	146.8	0.87	96	22
2	130.8	128.9	0.80	75	6
3	141.7	143.1	0.83	90	18
4	113.7	114.4	0.78	89	8
5	108.04	185.08	0.62	50	5

## Discussion

### Summary of Findings

In our methodology, we developed three DL classification models namely, VGG16, VGG19, and ResNet50. We selected ResNet50 model as the basic classification model for our framework as it outperforms the other two models on all the metrics.

For the image segmentation model, the overall performance for the 1,197 evaluation set is 88.5% based on the average DSC score which is close to accuracy of 87.2% reported in Dormer et al. (18) which involved the segmentation of the four cardiac chambers from CT images. The median (50% percentile) dice score of the proposed 2-dimensional UNet image segmentation model on the evaluation set is 0.93. Similarly, a recent effort in Chen et al. (19) that uses a more complex 3-dimensional UNet DL architecture for the segmentation of LA has also been shown to have a performance of 0.93 median dice score.

The RCA method (17) uses 100 images for obtaining the transformation matrix and warped segmentation mask pairs whose number increases with the number of images. In comparison, the mRCA method we proposed uses 29 images selected from a stack of 704 images by selecting images at 25 slice intervals. The choice of *p* = 5 patients and the selection of images at 25 slice intervals was to keep the sizes of the transformation matrices and the warped segmentation masks as small as possible (which reduces the time for computing the predicted DSC score) and at the same time ensure that the selected images are representative of the complex geometry of LA. This slice interval of 25 used for the mRCA method could be reduced further which, in turn, will increase the number of images and improve the accuracy of DSC score prediction at the expense increase in computation time. The mRCA method (mean dice score of 82%) for predicting DSC score shows far better performance compared to the RCA method (mean dice score of 52%) on testing dataset of 2,000 images but that was at the expense of increase in computation time. The process of classifying an image as containing LA, segmenting LA from the image and predicting its dice score takes 805 ms with RCA method while it takes 4,385 ms with mRCA method.

The degree of linear correlation between the label and predicted quality-controlled volumes, *R*^2^, is 0.968 for the study population of 337 patients. As neighboring slices are similar, for LAV estimation with QC, we have assessed the segmentation quality of slices at eight slice intervals for computing *q*_*mean*_, *q*_*perc*_, and *q*_*no*_. The number of slice intervals to estimate dice scores was chosen to be eight and that has worked for our volume computation problem where the interslice distance ranges from 2.2 to 4.5 mm. In practice, the number of slice intervals could be varied depending on the interslice distance settings of the acquisition machine. Generally, a small value could improve quality assessment at the expense of increase in computation time of LAV estimates.

The scores *q*_*mean*_, *q*_*perc*_, and *q*_*no*_ were used to develop a QC mechanism by imposing certain conditions on them in order to determine whether to accept or reject an estimated LAV computation. The approach correctly identified 9 out of 10 poor LAV estimations from a total of 337 patients as LAV estimation of poor quality (missing 1 out of 10). Of course, this condition may have worked for our task but, in practice, the constraint of this condition could be made more stringent or relaxed to suit a given problem or dataset. Moreover, the estimation of LAV without QC took ~23.32 s per patient. With QC, the estimation took 74.25 s per patient.

In addition, in relation to the Figures 7A,B, apart from the cases of the very small volumes (four out of nine) that would have been identified by simple thresholding even without the proposed quality control approach, the main reason that explains why our QC mechanism flagged these nine cases are that these patients have anatomical abnormalities that were unfamiliar to the ML models (that is, the dataset used for the training do not contain many of these abnormal cases and, therefore, the model does not generalize to these cases). Such abnormalities include large left atrial appendage, atypical atrial dimensions and geometries, and mispositioning of the pulmonary arteries.

For the classification and segmentation models, we have used the 150 patients (Dataset 1) and, for the volume computation, we have used the whole of the 337 patients. We note here that, in an attempt to make the classification and segmentation models more generalizable, we trained these models further using transfer learning approach (i.e., by training the models further but fixing certain part of the model weights) with the 151–337 datasets but these subsequent models were not superior to the initial models trained on Dataset 1. Furthermore, since model training was not carried out during volume estimation (that is, for example, we are not fitting a regression-like model where the input variables are the images and the output variable is the volume), it was meaningful to compare the computed volumes for the whole dataset of 337 patients using our approach with the results obtained by manual computation from an expert. In other words, our deep learning models are oblivious to the values of the volumes for all the 337 patients, and we are able to show that our approach is able to predict the volumes with high accuracy.

Researchers have successfully used DL for the segmentation and estimation of quantifiable measures of the different structures of the heart from CT images (18, 20). This set of algorithms include DL segmentation models which can find certain images challenging to segment owing to the considerable variation of cardiac anatomy among individuals. Importantly, these algorithms are not fully automated to compute quantifiable measures from the output of segmentation tasks. Algorithms that do, such as those given in Chen et al. (21), used *ad hoc* approaches that do not include mechanisms to detect or handle cases where image segmentation exercise fails which will inevitably affect the results of the computed quantities or the results of further analysis that are based on such quantities. This can be observed, for example, in our estimation of LAV in this study where DL without QC mechanism failed to correctly compute 10 out 337 LAV values. Practically, this could be costly in clinical decision support for diagnosis and treatments.

There are LAV estimation ML methods that have been proposed for other modalities. For example, automated volume estimation methods for the heart ventricles for cardiac magnetic resonance (CMR) images, such as Bayesian method (7), method of linear support vector machines (8), and method of multi-scale deep belief networks and regression forests (9), have been proposed that are capable of estimating the volumes directly when given the set of images containing LA and without the image classification or segmentation processes. Despite the promise of these approaches, they have only been developed with very limited amount of data [e.g., 56 subjects in each of Wang et al. (7) and Zhen et al. (9) and 58 subjects in Afshin et al. (8)], meaning that, the generalizability and evaluation of the methods to large datasets have not been reported.

QC of medical images involves identifying those images with problems that resulted from the image acquisition process, image reconstruction algorithm or even in a step of the image processing pipeline. QC tasks are important to ensure the usability of these images. QC assessment *via* visual inspection is highly subjective, time consuming, and infeasible in large-scale image acquisition or analysis settings. Methods that have been developed for QC assessment of medical images include the estimation of image noise level (22, 23), random forest supervised learning approaches (24, 25), simple label voting scheme (26), probabilistic schemes (27, 28), and Bayesian neural networks learning (29–31). Many of these methods are inherently specific to the tissues being segmented or to the modalities used for image acquisition.

With advancement in the applications of AI in medical imaging, it is important to have QC methods that can assess the quality of the analyses being carried out by AI techniques. Current studies on this subject have only focused on the image segmentation part of the process. Researchers, such as in Valindria et al. (16), Robinson et al. (17), Biasiolli et al. (32), and Hann et al. (33) reduce the problem to developing classifiers for estimating Dice Similarity Coefficient (DSC), one of the common metrics for image segmentation accuracy, in the absence of manual segmentation as ground truths. The classifiers are often trained using random forests (16, 32) or convolutional neural network (16, 33) on a fixed number of space (k-space) of segmentation features with cases where ground truths are already known. In Robinson et al. (17), QC of image segmentation task of CMR images was proposed. The method used the RCA technique (16, 17) to predict the DSC score. QC is carried out by classifying the segmentation mask of an image as good quality if the accuracy estimate is above a pre-defined threshold value, and as poor quality otherwise.

The existing DL algorithms dealing with automated segmentation of CT images do not come with segmentation QC mechanisms. This is an important setback in practice as it makes the algorithms unsuitable for fully automated analysis tasks in clinical applications. Moreover, in a problem like LAV estimation in which segmentation is part of the process and not itself the end goal of the analysis, the errors at the segmentation level add up cumulatively in the computation of the volumes. Thus, a QC method for DL-based segmentation models that can identify cases that are difficult or inaccurate as presented in this paper will be immensely useful.

### Limitation

Our models were trained on data from a single hospital system, limiting their generalizability. Data augmentation may have improved the chance that performance may not deteriorate significantly from datasets from elsewhere. It would therefore be worthwhile assessing the performance of these models using an external dataset. Accordingly, the performance of the models may then be improved, if needed, with training on heterogenous multi-center datasets. In addition, the framework we have proposed is not limited by data from a single hospital. We believe our approach is generalizable for building models with multi-center datasets with little or no modification. Importantly, the cases that are flagged as inaccurate LAV estimates in our approach could be interpreted as samples where there is little dataset on which the models have learnt from. The issue can then be addressed by providing training data that covers those areas of interest. This approach could well-prove useful in the future given the current trend in AI research is toward developing data-centric AI algorithms (34).

### Future Work

The mRCA could be further optimized to reduce computation time, possibly by reducing the sizes of the transformation matrices and the warped segmentation masks. The quality assessment method is not based on checking the segmentation quality of every slice, it should therefore be worthwhile exploring the optimal interslice interval that will reduce computational time without degrading the quality of LAV prediction. Although our framework focused on LAV estimation with QC for CT images, the framework can be adapted to estimate volumes of other tissues and for images from other modalities.

## Conclusion

We proposed a novel comprehensive framework that consists of DL models and computational methods for LAV estimation. Although the individual DL model architectures in this work are not new, putting them together in a single framework with a novel QC mechanism as we have done is novel and more clinically useful. The framework provides an efficient and robust strategy for QC assessment of the accuracy for DL-based image segmentation and volume estimation tasks. It therefore efficiently fully automates the process of estimating LAV and is able to flag cases where it fails to accomplish high quality prediction, perhaps, better described as a framework that allows DL models to “grade their own homework.” The framework we have proposed in this paper will be highly appreciated by clinicians for carrying out LAV estimation, particularly for large cohorts and high-throughput applications, and practitioners involved in developing digital solutions around CCT image applications such as CCT image acquisition that will allow better analysis results.

## Data Availability Statement

The datasets presented in this article are not readily available because restrictions apply to the availability of these raw data, which were used under license for the current study from Georgetown University Institutional Review Board, and so are not publicly available. Generated anonymized dataset are however available from the authors upon reasonable request and with permission of Georgetown University Institutional Review Board. Requests to access the datasets should be directed to Jose D. Vargas, jose.vargas@nih.gov.

## Ethics Statement

The studies involving human participants were reviewed and approved by Georgetown University Institutional Review Board (STUDY-0400, approved 7/20/2017). Dataset were fully anonymized. The patients/participants provided their written informed consent to participate in this study.

## Author Contributions

MA, MB, JV, and SP conceived the idea and contributed to the analysis. MB, JV, FZ, ATa, ATh, PB, and MS developed the contouring method. MA led on the machine learning methodology and the main mathematical and statistical analysis of CT data and images. MB and JV advised on cardiac CT analysis and validation. MA, AL, and SP advised on data governance and computing infrastructure. MA drafted the first version of the manuscript. SP and JV provided overall supervision. All authors contributed to the content, the writing of the final version, and provided critical feedback.

## Funding

MA and SP acknowledge support from the CAP-AI programme (led by Capital Enterprise in partnership with Barts Health NHS Trust and Digital Catapult and funded by the European Regional Development Fund and Barts Charity) and Health Data Research UK (HDR UK—an initiative funded by UK Research and Innovation, Department of Health and Social Care (England) and the devolved administrations, and leading medical research charities; www.hdruk.ac.uk). SP acknowledges support from the National Institute for Health Research (NIHR) Biomedical Research Center at Barts, from the SmartHeart EPSRC programme grant (www.nihr.ac.uk; EP/P001009/1) and the London Medical Imaging and AI Center for Value-Based Healthcare. SP has received funding from the European Union's Horizon 2020 research and innovation programme under grant agreement No. 825903 (euCanSHare project).

## Conflict of Interest

The authors declare that the research was conducted in the absence of any commercial or financial relationships that could be construed as a potential conflict of interest.

## Publisher's Note

All claims expressed in this article are solely those of the authors and do not necessarily represent those of their affiliated organizations, or those of the publisher, the editors and the reviewers. Any product that may be evaluated in this article, or claim that may be made by its manufacturer, is not guaranteed or endorsed by the publisher.

## Abbreviations

AF: atrial fibrillation
AI: artificial intelligence
CA: catheter ablation
CCT: cardiac CT
CMR: cardiac magnetic resonance
CT: computed tomography
DL: deep learning
DSC: dice similarity coefficient
LA: left atrium
LAV: LA volume
ML: machine learning
mRCA: modified RCA
PV: pulmonary vein
QC: quality control
RCA: reverse classification accuracy.
